# Do the etiology of hyponatremia and serum sodium levels affect the length of hospital stay in geriatric patients with hyponatremia?

**DOI:** 10.5937/jomb0-29914

**Published:** 2022-02-02

**Authors:** Salih Baser, Cakmak Nuray Yılmaz, Emin Gemcioglu

**Affiliations:** 1 Yıldırım Beyazıt University, Faculty of Medicine, Ankara City Hospital, Department of Internal Medicine, Ankara, Turkey; 2 Ankara City Hospital, Department of Internal Medicine, Ankara, Turkey

**Keywords:** hyponatremia, geriatrics, length of stay, hiponatremija, gerijatrija, dužina boravka

## Abstract

**Background:**

Hyponatremia can lead to a prolonged hospital stay and increased morbidity and mortality rates in geriatric patients. This study aimed to evaluate the effects of hyponatremia etiology and serum sodium (Na) levels on hospitalisation time in geriatric patients hospitalised due to hyponatremia.

**Methods:**

The demographic characteristics, laboratory data, etiology of hyponatremia, and length of hospital stay were retrospectively recorded for 132 patients over 65 years of age who were hospitalised for hyponatremia.

**Results:**

Of the 132 patients, 90 were female (68.2%), and 42 were male (31.8%). The serum Na levels of 66 (50%) patients were <120 mmol/L, those of 64 (48.5%) patients were 120-129 mmol/L, and those of two (1.5%) patients were >130 mmol/L. One hundred nine (82.6%) patients had hypoosmolar hyponatremia, 14 (10.6%) patients had isoosmolar hyponatremia, and nine (6.8%) patients had hyperosmolar hyponatremia. Also, 19.7% of the patients were hypovolemic, 37.9% were euvolemic, and 42.4% were hypervolemic. Hyponatremia etiology was congestive heart failure in 38 (28.8%) patients, syndrome of inappropriate antidiuretic hormone in 29 (22.0%) patients, gastrointestinal fluid loss in 24 (18.2%) patients, renal pathologies in 20 (15.2%) patients, the presence of drugs in 20 (15.2%) patients, and hypocortisolemia in one (0.8%) patient. The mean length of hospital stay for the patients was five (1-60) days. There was no statistically significant difference between the lengths of hospital stay based on hyponatremia etiology and serum Na levels (p=0.861 and p=0.076). It was observed that the lengths of stay for patients who developed hyponatremia during their hospitalisation in various clinics were longer than those for patients who presented to the emergency department (p<0.001).

**Conclusions:**

In this study, it was determined that the length of hospital stay did not change with the etiology of hyponatremia and serum Na level at the time of admission, but patients who developed hyponatremia during their hospitalisation had longer hospitalisation times.

## Abstract (sr)

**Uvod:**

Hiponatremija može dovesti do produženog boravka u bolnici i povećane stope morbiditeta i mortaliteta kod gerijatrijskih pacijenata. Cilj ove studije bio je da se procene efekti etiologije hiponatremije i nivoa natrijuma (Na) u serumu na vreme hospitalizacije kod gerijatrijskih pacijenata hospitalizovanih zbog hiponatremije.

**Metode:**

Retrospektivno su zabeležene demografske karakteristike, laboratorijski podaci, etiologija hiponatremije i dužina boravka u bolnici za 132 pacijenta starijih od 65 godina koji su hospitalizovani zbog hiponatremije.

**Rezultati:**

Od 132 pacijenta, 90 su bile žene (68,2%), a 42 muškarci (31,8%). Nivoi Na u serumu 66 (50%) pacijenata su bili < 120 mmol/L, kod 64 (48,5%) pacijenata su bili 120-129 mmol/L, a kod dvoje (1,5%) pacijenta > 130 mmol/L. Sto devet (82,6%) pacijenata je imalo hipoosmolarnu hiponatremiju, 14 (10,6%) bolesnika je imalo izoosmolarnu hiponatremiju, a devetoro (6,8%) pacijenata je imalo hiperosmolarnu hiponatremiju. Takođe, 19,7% pacijenata bilo je hipovolemično, 37,9% euvolemično, a 42,4% hipervolemično. Kod 38 (28,8%) pacijenata etiologija hiponatremije je bila kongestivna srčana insuficijencija, kod 29 (22,0%) pacijenata sindrom neodgovarajućeg antidiuretskog hormona, gubitak gastrointestinalne tečnosti kod 24 (18,2%) pacijenata, bubrežne patologije kod 20 (15,2%) pacijenata, prisustvo lekova kod 20 (15,2%) pacijenata, a kod jednog (0,8%) pacijenta hipokortizolemija. Prosečna dužina boravka u bolnici za pacijente bila je pet (1-60) dana. Nije bilo statistički značajne razlike između dužine boravka u bolnici na osnovu etiologije hiponatremije i nivoa Na u serumu (p = 0,861 i p = 0,076). Uočeno je da su dužine boravka kod pacijenata koji su razvili hiponatremiju tokom hospitalizacije u raznim klinikama bile duže nego kod pacijenata koji su došli na odeljenje hitne pomoći (p < 0,001).

**Zaključak:**

U ovoj studiji je utvrđeno da se dužina boravka u bolnici nije menjala sa etiologijom hiponatremije i nivoa Na u serumu u trenutku prijema, ali su pacijenti koji su razvili hiponatremiju tokom hospitalizacije imali duže vreme hospitalizacije.

## Introduction

Hyponatremia is the most common electrolyte abnormality observed in clinical practice. It can be seen in about 30% of hospitalised patients and can lead to a wide range of clinical symptoms, from asymptomatic to severe and even life-threatening [1] [2].

In order to determine the diagnosis and treatment in patients presenting with hyponatremia, grouping is performed according to patients' serum osmolality and volume status. Serum osmolality is grouped as hypoosmolar at <280 mmol/kg, isoosmolar at 280-295 mmol/kg, and hyperosmolar at >295 mmol/kg, with a further categorisation of hypovolemic, euvolemic, or hypervolemic hyponatremia according to volume status. Symptoms depend on the severity and duration of the hyponatremia. Acute hyponatremia is defined by the onset of symptoms within 48 h. Patients with acute hyponatremia develop neurologic symptoms caused by cerebral edema due to water movement into the brain. These may include seizures, impaired mental status, or coma and death. Hyponatremia developing over longer than 48 h is considered chronic hyponatremia. Treatment depends on the acute or chronic onset, the patient's volume status, and the severity and nature of the symptoms.

Age is a strong independent risk factor for hyponatremia, and older patients constitute a highrisk group for its occurrence [2] [3] [4] [5]. Symptoms such as nausea, vomiting, headache, stupor, coma, and seizures are associated with acute hyponatremia and fatigue, cognitive impairment, and gait defects are associated with chronic hyponatremia; also, falls, poor bone quality (e.g., osteoporosis), and negative effects of fractures are more frequent and severe in geriatric patients [6] [7] [8] [9] [10]. Hyponatremia also prolongs the hospitalisation time remarkably and increases the cost of medical care substantially [11]. It is unknown whether there is a relationship between the etiology of hyponatremia or initial serum sodium (Na) levels and the length of hospital stay. This study intended to evaluate the clinical features, hyponatremia etiologies, and hospitalisation durations of hyponatremic patients over 65 years of age and determine whether there is a relationship between hyponatremia etiology and the length of hospital stay.

## Materials and Methods

### Study Population

In this study, 132 patients aged 65 years who were hospitalised in our clinic due to hyponatremia were evaluated retrospectively. Patients with complete data were included in the study. Patients aged <65 years and those with hyperglycemia, hyperlipidemia, or paraproteinemia that could cause pseudohyponatremia were excluded from the study. The approval of the local ethics committee was obtained (18.09.2019/94).

The demographic characteristics of the patients (age, gender) and their places of presentation (emergency room, other clinics), presenting complaints (nausea, vomiting, confusion, seizure, fever, dyspnea, edema, general condition disorder, fatigue, anorexia), physical examination findings, volume statuses (hypovolemic, euvolemic, hypervolemic), hyponatremia etiologies, treatments (hypertonic saline, isotonic saline, water restriction, diuretics, ultrafiltration), and laboratory data (glucose, urea, creatinine, serum osmolality, Na, potassium (K), urine Na, urine osmolality, hemoglobin, thyrotropin (TSH), and serum cortisol levels) were recorded. The normal serum Na range is 135-145 mmol/L, serum osmolality is 275-293 mmol/kg serum water, and urine osmolality is 500-850 mmol/kg water. Hyponatremia is defined as an Na of <135 mmol/L, and severe hyponatremia is defined as serum Na of <120 mmol/L.

### Statistical Analysis

The IBM SPSS 21.0 statistical software package for Windows was used for the statistical analysis of the data. For all data, the normality assumption was evaluated via the Shapiro-Wilk test. Numerical data are indicated by median (minimum-maximum), and categorical data are indicated by numbers (percentage). The Mann-Whitney U test was used to compare numerical data between two groups, and the Kruskal-Wallis test was used to compare more than twogroups. Values of p<0.05 were considered statistically significant.

## Results

Of the 132 patients, 90 were female (68.2%), 42 were male (31.8%), and the mean age was 74.97±7.14 years. Severe hyponatremia (Na of <120 mmol/L) was detected in 66 (50%) patients.

While 97 (73.5%) patients presented to the emergency department with complaints related tohyponatremia, 35 (26.5%) patients were found to have developed hyponatremia during their hospitalisation in various clinics. Dyspnea and edema were observed in 28 (21.2%) patients, nausea/vomiting in 26 (19.7%) patients, confusion in 26 (19.7%) patients, fatigue and anorexia in 15 (11.4%) patients, seizures in eight (6.1%) patients, fever in eight (6.1%) patients, and general condition disorder in five (3.8%) patients, whereas 16 (12.1%) patients were asymptomatic. The median systolic blood pressure of the patients was 120 (68–250) mmHg, and the mean diastolic blood pressure was 70 (39–130) mmHg. The physical examination findings of the patients at the time of admission are presented in Table 1. Hypovolemic hyponatremia was detected in 26 (19.7%) patients, euvolemic hyponatremia in 50 (37.9%) patients, and hypervolemic hyponatremia in 56 (42.4%) patients.

**Table 1:** Demographic features, complaints, and physical examination findings of the patients

	n (%)
Gender	
Female	90 (68.2)
Male	42 (31.8)
Place of application	
Emergency department	97 (73.5)
Different clinics	35 (26.5)
Complaints	
Dyspnea/edema	28 (21.2)
Nausea/vomiting	26 (19.7)
Confusion	26 (19.7)
Asymptomatic	16 (12.1)
Fatigue/anorexia	15 (11.4)
Seizures	8 (6.1)
Fever	8 (6.1)
General condition disorder	5 (3.8)
Physical examination	
Rales in the lung	
Present	42 (31.8)
Absent	90 (68.2)
Ascites	
Present	5 (3.8)
Absent	127 (96.2)
Pretibial edema	
Present	36 (28.3)
Absent	91 (71.7)
Volume	
Hypovolemic	26 (19.7)
Euvolemic	50 (37.9)
Hypervolemic	56 (42.4)

Serum Na level was 119.50 (99-131) mmol/L at the time of admission, 125 (105-139) mmol/L at the 24th hour of treatment, and 128.50 (108-144) mmol/L at the 48th hour of treatment (p<0.001). The serum Na levels of 66 (50%) patients were <120 mmol/L, those of 64 (48.5%) patients were 120-129 mmol/L, and those of two (1.5%) patients were >130 mmol/L. One hundred nine (82.6%) patients had hypoosmolar hyponatremia, 14 (10.6%) patients had isoosmolar hyponatremia, and nine (6.8%) patients had hyperosmolar hyponatremia (Table 2).

**Table 2:** Laboratory values of the patients TSH: Thyrotropin, Na: sodium

	Median (min–max)
Glucose (mmol/L)	5.44 (3.77–6.72)
Urea nitrogen (mmol/L)	7.32 (0.51–36.03)
Creatinine (mmol/L)	79.56 (13.26–739.9)
Potassium (mmol/L)	4.40 (2.30–6.70)
TSH (mIU/L)	1.1 (0.92–5.2)
Serum cortisol (nmol/L)	317.2 (110.3–717.2)
Hemoglobin (g/L)	112 (62–179)
Serum Na level at the time of admission (mmol/L)	119.50 (99–131)
Serum Na level at the 24th hour of treatment (mmol/L)	125 (105–139)
Serum Na level at the 48th hour of treatment (mmol/L)	128.50 (108–144)
Serum osmolality (mmol/kg)	259 (212–309)
Urine osmolality (mmol/kg)	224 (36–782)
Urine Na (mmol/L)	45.5 (4–321)
Na groups	n (%)
<120 mmol/L<br>120 –129 mmol/L<br>130 –135 mmol/L	66 (50)<br>64 (48.5)<br>2 (1.5)
Osmolality groups	n (%)
Hypoosmolar<br>Isoosmolar<br>Hyperosmolar	109 (82.6)<br>14 (10.6)<br>9 (6.8)

The etiology of hyponatremia was congestive heart failure in 38 (28.8%) patients, syndrome of inappropriate antidiuretic hormone (ADH) secretion (SIADH) in 29 (22.0%) patients, gastrointestinal fluid loss in 24 (18.2%) patients, renal pathologies in 20 (15.2%) patients, the presence of drugs in 20 (15.2%) patients, and hypocortisolemia in one (0.8%) patient. In 51.7% of patients with SIADH, the cause was an infection, with the most common (66.7%) reason being pneumonia.

Sixty-eight (51.5%) patients were treated with hypertonic saline, 20 (15.2%) with isotonic saline, 24 (18.2%) with water restriction and diuretics, and 20 (15.2%) with only water restriction. It was observed that seven (5.3%) patients required ultrafiltration.

The mean length of hospital stay for the patients was 5 (1-60) days. There was no statistically significant difference between the lengths of hospital stay in terms of hyponatremia etiologies (p=0.861). In addition, serum Na levels at the time of presentation did not show a statistically significant difference in terms of hyponatremia etiologies (p=0.065). It was observed that the lengths of hospital stay and serum Na levels at the time of presentation were similar in female and male patients (p=0.440 and p=0.230). In addition, there was no statistically significant difference between the duration of hospital stay in patients with serum Na levels of <120 mmol/L and 120-129 mmol/L at the time of admission (p=0.076). It was observed that the lengths of hospital stay for patients who developed hyponatremia during their hospitalisation in various clinics was longer than those of patients who presented to the emergency department (p<0.001), but serum Na levels were higher in patients who developed hyponatremia during hospitalisation (p<0.001) (Table 3).

**Table 3:** Duration of hospitalisation and sodium levels at the time of patients’ admission according to gender, etiology, place of application, and patients’ duration of hospitalisation according to sodium groups Na: Sodium, SIADH: syndrome of inappropriate antidiuretic hormone secretion

	Duration of hospitalisation	p	Na levels at the time of admission	p
Gender				
Female	4 (1–60)	0.440	119<br> (101–131)	0.230
Male	7 (1–43)		121<br> (99–129)	
Etiology				
Congestiveheart failure	4 (1–30)	0.861	119<br>(99–128)	0.065
SIADH	6 (1–59)		121<br>(109–129)	
Gastrointestinalfluid loss	4 (1–36)		119<br>(101–126)	
Renalpathologies	7 (1–60)		122.5<br(105–129)	
Drugs	4 (1–43)		120.5<br(108–131)	
Place of application				
Emergencydepartment	3 (1–59)	<0.001	118<br>(99–130)	<0.001
Different clinics	10 (2–60)		124<br>(111–131)	
Na groups				
<120 mmol/L	4 (1–59)	0.076	-	-
120–129	6 (1–60)			

## Discussion

Hyponatremia is the most common electrolyte disorder in hospitalised patients and society. Hyponatremia prevalence in society is ~8%, and this prevalence increases significantly with age [3] [4]. Hyponatremia is reported to be associated with an increased risk of mortality and poor prognosis in older individuals [3] [12].

The higher rate of hyponatremia in the elderly is related to the deterioration of the water excretion capacity associated with aging and the more frequent exposure of this group to drugs and diseases associated with hyponatremia [2] [13]. The decrease in the glomerular filtration rate due to aging causes impaired water excretion capacity. In addition, the decrease in intrarenal prostaglandin production seen at older ages may cause impaired water excretion capacity [14]. Another factor contributing to hyponatremia in this group is the fact that the age-related decrease in total body water percentage causes further fluctuations in serum Na concentration. Higher sensitivity to osmotic stimuli can be seen in the geriatric population [15] [16]. Elderly individuals frequently use drugs known to cause hyponatremia (such as thiazide diuretics, selective serotonin reuptake inhibitors, and nonsteroidal anti-inflammatory drugs), and they often suffer from diseases that may be associated with hyponatremia (for example, diabetes mellitus, infections, heart failure, liver diseases, malignancies, and endocrinopathies) [17] [18]. Many elderly patients with hypertension or heart failure maintain a low-salt diet, which can cause a low serum Na concentration. In this population, a decrease in protein intake due to overlapping diseases may play a role in the development of hyponatremia by impairing water excretion [19] [20].

Diuretics and SIADH are among the most common causes of hyponatremia in the elderly [20] [21]. In one prospective study that included only elderly hospitalised patients, the most common causes of hyponatremia were SIADH and diuretics. In the same study, the two most common causes of SIADH were lower respiratory tract infection and stroke [22]. In the study of Chatterjee et al. [23], gastrointestinal fluid loss, cerebrovascular accident, and pulmonary sepsis were found to be the most frequent causes of hyponatremia. In the work of Babaliche et al. [24], SIADH was also the most common cause of hyponatremia in 46% of patients, followed by renal pathologies in 13%, gastrointestinal compromise in 11%, cardiac causes in 10%, cirrhosis in 10%, and drugs in 10%. In addition, Ishikawa et al. [25] reported that 40% of patients presenting with hyponatremia aged 65 and above had hypothalamic-pituitaryadrenal dysfunction. Although congestive heart failure was reported in other studies as a less common cause of hyponatremia than diuretics and SIADH, the most common cause of hyponatremia in our study was congestive heart failure, the second most common cause was SIADH [23] [24]. Contrary to the study of Ishikawa et al. [25], hyponatremia due to hypopituitarism was very rare in our study group. This may be because patients with hypopituitarism are asymptomatic for long periods, and their need for hospitalisation is less than those of other patients. Because only hospitalised patients were included in our study, the rate of hypopituitarism may be lower than expected.

The importance of early recognition of hyponatremia and prompt intervention is critical [26]. In a large multicenter trial with 151,486 patients, it was shown that all types and grades of dysnatremias were related to increased risk-adjusted and raw hospital mortality rates. The odds ratios for mild, moderate, and severe hyponatremia were 1.32, 1.89, and 1.81, respectively [27]. Moreover, in addition to mortality, hyponatremia prolongs the hospitalisation time remarkably and increases medical care costs [11]. In our study, the length of hospital stay due to hyponatremia was observed to be 5 (1-60) days, and this duration did not change according to the etiology of hyponatremia or the patient's gender or initial serum Na levels. It was observed that patients who applied to the emergency department had lower Na levels but shorter hospital stays than patients who developed hyponatremia during their hospitalisation in other clinics.

In their study, including 100 patients with moderate to severe hyponatremia who were monitored in the intensive care unit, Babaliche et al. [24] reported that 59% of the patients were male and 41% were female, with a slight dominance of the male gender. In the work of Sood et al. [28], the male-to-female ratio was 1.25:1. In other studies in the literature, male gender dominance is observed in patients with hyponatremia [23] [29]. Contrary to these studies, in our study, 68.2% of patients with hyponatremia were female. Since our study consists of randomly recruited patients for a certain period of time, the gender result may be due to this.

In the study of Sood et al. [28], including 106 hyponatremic patients, 90% were hypoosmolar, 9% hyperosmolar, and 1% were isoosmolar, while 40% were euvolemic, 31% were hypervolemic, and 29% were hypovolemic. In the study of Chatterjee et al. [23], 50.74% of the patients were euvolemic, 26.86% were hypervolemic, and 22.4% were hypovolemic, while in the study of Babaliche et al. [24], 50% were euvol emic, 33% were hypervolemic, and 17% were hypovolemic. In our study, 42.4% of the patients were hypervolemic, 37.9% were euvolemic, and 19.7% were hypovolemic while 82.6% had hypoosmolar hyponatremia, 10.6% had isoosmolar hyponatremia, and 6.8% had hyperosmolar hyponatremia.

In their study, Sood et al. [28] reported that 42% of patients had severe hyponatremia, 48% had moderate hyponatremia, and 10% mild hyponatremia. Similarly, in our study, severe hyponatremia was detected in 50% of the hospitalised geriatric patients. It was observed that Na levels were 120-129 mmol/L in 48.5% and 130-135 mmol/L in 1.5% of the patients.

Pillai et al. [30] observed that among intensive care unit admissions, the symptoms attributed to hyponatremia included nausea (69.3%), malaise (80%), drowsiness (61.3%), confusion (41.3%), lethargy (24%), frequent falls (1.3%), convulsions (2.7%), altered sensorium (41.3%), and delirium (9.3%). Krishnamurthy and Srinivas [31] reported that the symptoms found in hyponatremia patients were vomiting (29.6%), giddiness (2.4%), altered sensorium (8.5%), headache (9.2%), chest pain (6.4%), generalized weakness (8.4%), fever (12.3%), cough (15.2%), loss of consciousness (0.7%), nausea (22.5%), loose stools (5%), increased fatigability (10.4%), breathlessness (17.8%), abdominal pain (8.8%), difficulty in micturition (0.9%), lower limb swelling (3.6%), and seizures (6.4%) (31). In our study, dyspnea and edema were observed in 28 (21.2%) patients, nausea/vomiting in 26 (19.7%) patients, confusion in 26 (19.7%) patients, fatigue and anorexia in 15 (11.4%) patients, seizures in eight (6.1%) patients, fever in eight (6.1%) patients, and general condition disorder in five (3.8%) patients, whereas 16 (12.1%) patients were asymptomatic.

In acute symptomatic hyponatremia, hypertonic saline solution is commonly used to acutely increase serum Na levels and prevent serious neurological symptoms. Hypovolemic hyponatremia is treated with adequate fluid resuscitation to reduce ADH secretion stimulation. Normal saline is often used to suppress the hypovolemic stimulus that causes ADH release [32] [33]. In patients with SIADH, careful administration of hypertonic fluids may be required, along with discontinuation of suspicious drugs and reduced water consumption. In these cases, furosemide can also be administered to prevent circulatory overload, especially if elderly patients have concomitant cardiac dysfunction. Furosemide increases free water excretion and leads to higher serum Na. Our study observed that 51.5% of the patients were treated with hypertonic saline, 15.2% with isotonic saline, 18.2% with water restriction and diuretics, and 15.2% with only water restriction. It was also observed that 5.3% of the patients required ultrafiltration.

In conclusion, there is an increasing tendency for hyponatremia to occur with increased age, comorbidities, and the use of drugs. In our study, congestive heart failure and SIADH were determined to be the most common causes of hyponatremia in geriatric patients. Nausea, vomiting, and dyspnea were the most common symptoms. It was determined that the length of hospital stay did not change with the etiology of hyponatremia, gender, or serum Na level at the time of admission. However, patients who developed hyponatremia during their hospitalisation had longer hospitalisation times.

## Dodatak

### Funding

The author(s) received no financial support for the research, authorship, and/or publication of this article.

### Conflict of interest statement

All the authors declare that they have no conflict of interest in this work.
